# Unique ultrastructural organization of human rod photoreceptors

**DOI:** 10.1038/s42003-025-07473-6

**Published:** 2025-01-16

**Authors:** Tylor R. Lewis, Natalia V. Klementieva, Sebastien Phan, Carson M. Castillo, Keun-Young Kim, Lauren Y. Cao, Mark H. Ellisman, Vadim Y. Arshavsky, Oleg Alekseev

**Affiliations:** 1 Department of Ophthalmology, Duke University School of Medicine, Durham, NC USA; 2 Department of Ophthalmology and Visual Sciences, University of Alabama at Birmingham, Birmingham, AL USA; 3 National Center for Microscopy and Imaging Research, Department of Neurosciences, School of Medicine, University of California San Diego, La Jolla, CA USA; 4 Department of Pharmacology and Cancer Biology, Duke University School of Medicine, Durham, NC USA

**Keywords:** Retina, Retina

## Abstract

Rod and cone photoreceptor cells are specialized neurons responsible for transforming the information reaching the eyes in the form of photons into the language of neuronal activity. Rods are the most prevalent photoreceptor type, primarily responsible for light detection under conditions of limited illumination. Here we demonstrate that human rods have a morphological organization unique among all described species, whereby the cell soma extends alongside the light-sensitive outer segment compartment to form a structure we have termed the “accessory inner segment.” These structures have two striking features: they are reinforced by a massive microtubular cytoskeleton and contain electron-dense adhesions that mediate their attachment to outer segments. Given that the spacing of human rod photoreceptors is sparser than in most other species, the accessory inner segment likely provides mechanical support to the closely apposed outer segment. This discovery expands our understanding of the human retina and directs future studies of human photoreceptor function in health and disease.

## Abstract (web summary)

Ultrastructural analysis of human retinas reveals that the inner segment of human rods extends alongside the outer segment to form the “accessory inner segment,” a unique microtubule-based structure that forms adhesions to the outer segment.

## Introduction

Light detection confers a substantial evolutionary advantage to all life forms, from cyanobacteria to humans^1^. In most species, it is accomplished by photoreceptor neurons, which contain highly specialized cellular compartments enriched in visual pigments and downstream signaling proteins. This anatomical arrangement confers their remarkable light sensitivity, including single photon detection^2^.

In vertebrate photoreceptors, the light-sensitive outer segment (OS) compartment is a modified primary cilium consisting of hundreds of flattened membrane discs enriched in visual pigments. The OS emanates from the apical end of the photoreceptor cell body, called the inner segment (IS), which performs housekeeping functions of the cell. At the opposite end of the cell is located the synaptic terminal, which propagates visual signals to downstream neurons. This general anatomical organization has been described for a wide variety of species (reviewed in refs. ^3–5^), including humans (e.g., refs. ^6–10^). Here, we revisited the ultrastructure of human rod photoreceptors and uncovered a unique anatomical feature overlooked in previous studies.

## Results

### Human rod photoreceptors have an accessory inner segment

**Fig. 1:**
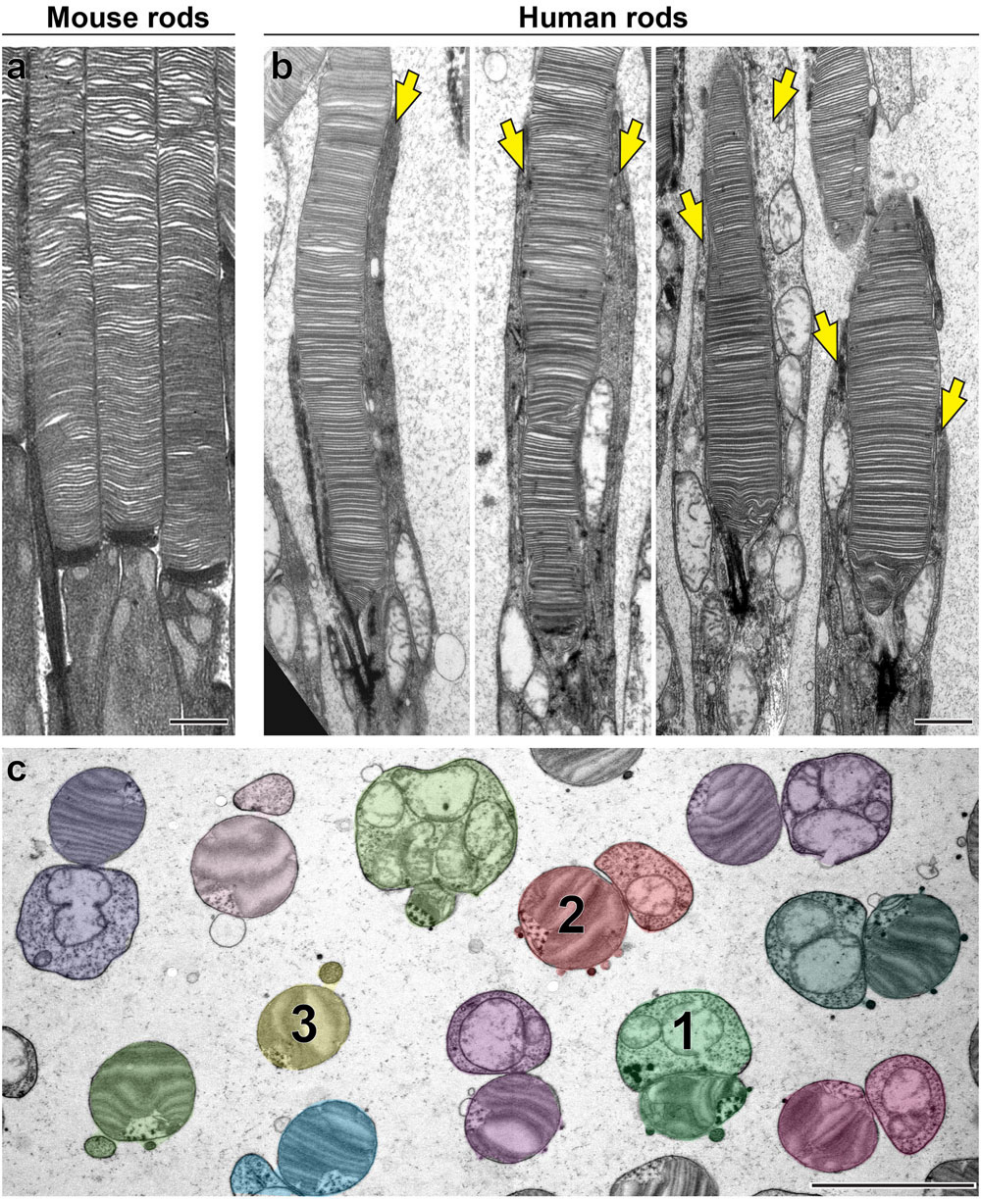
Human rod photoreceptors have an accessory inner segment. TEM images of rod photoreceptors from longitudinally sectioned mouse (**a**) and human (**b**) retinas. **c** A low-magnification TEM image of tangentially sectioned human retina. Rods containing a paired aIS and OS are pseudo-colored. Three numbered cells are sectioned at different axial positions ranging from proximal (example 1) to intermediate (example 2) and distal (example 3). Yellow arrows indicate aIS. Scale bars: 1 µm (**a**, **b**); 5 µm (**c**).

Ultrastructural analysis of longitudinally sectioned healthy human retinas using transmission electron microscopy (TEM) revealed an immediate striking observation that sets human rod photoreceptors apart from those of essentially all other previously characterized vertebrate species. In other species, there is a clear vertical distinction between the photoreceptor IS and OS, with the OS located directly apical to the IS^11^ (Fig. 1a). In contrast, a large portion of the IS in human rods extends alongside the OS to form structures which we have termed the “accessory inner segment” (aIS; Fig. 1b).

**Fig. 2:**
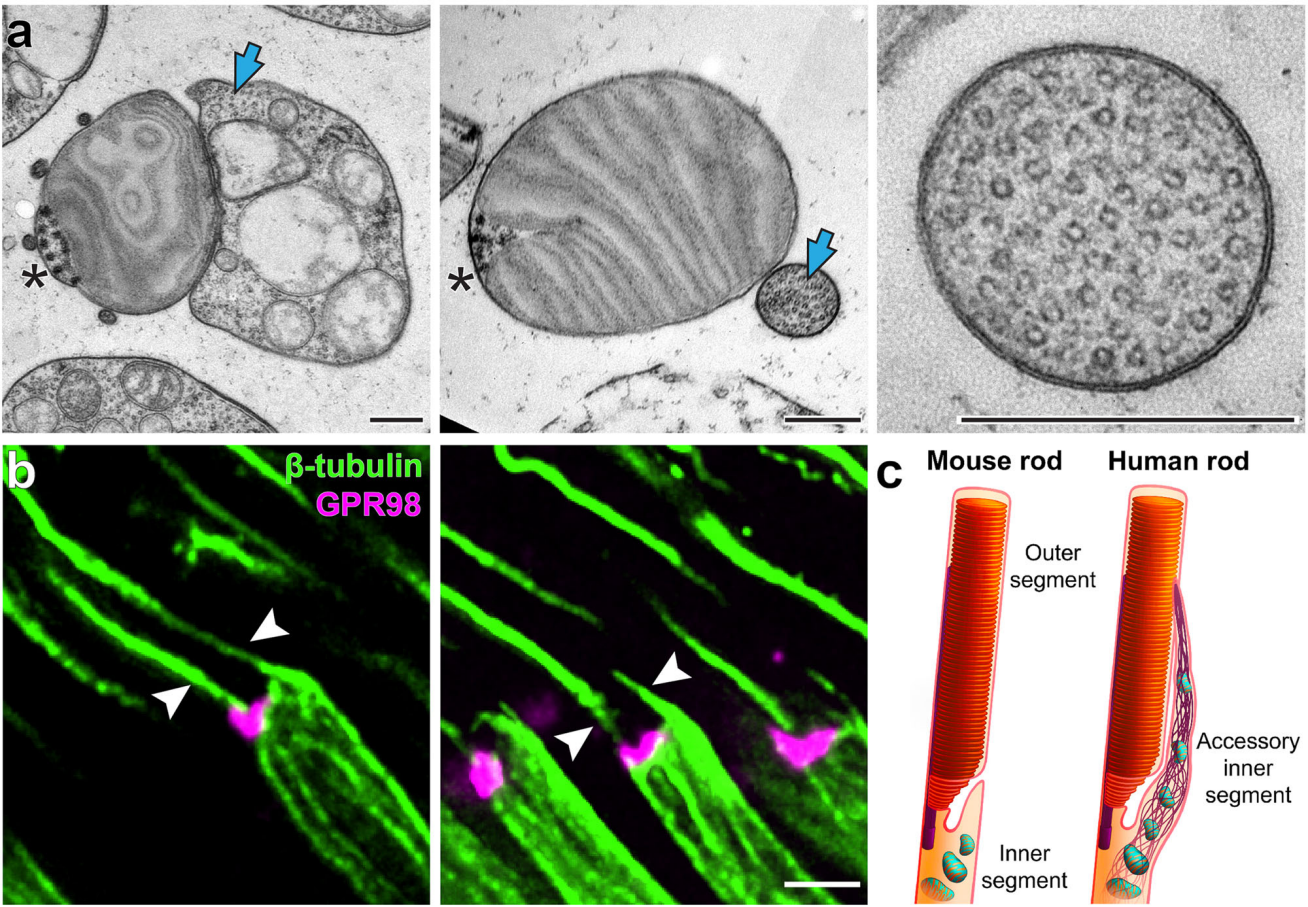
The accessory inner segment contains microtubules. **a** TEM images of human rods tangentially sectioned at intermediate (left panel) and distal (middle panel) positions. Blue arrows indicate bundles of microtubules inside the aIS. The right panel shows a magnified image of the distal aIS devoid of mitochondria and tightly packed with microtubules. Asterisks indicate the ciliary axoneme. **b** Immunofluorescence images of rods from longitudinally sectioned human retina, in which microtubules (green) and the connecting cilium region (magenta) are labeled with antibodies against β-tubulin and GPR98, respectively. Arrowheads indicate microtubules emanating from opposite sides of the OS base within an individual rod. **c** Schematic representation of the accessory inner segment of human rod photoreceptors. The image on the left represents the typical vertical arrangement of the IS and OS in most studied species, such as the mouse. The image on the right illustrates the new observation from this study that human rods have an aIS extending from the IS alongside the OS. The base of the aIS is comparable in width to the OS and contains mitochondria as well as a microtubule-based cytoskeleton. The apical portion of the aIS tapers in diameter and contains a dense bundle of microtubules. For ease of visualization, the smaller microtubule-containing processes, as well as inner segment microtubules, are not shown. Scale bars: 0.4 µm (**a**); 2 µm (**b**).

To further investigate the structure and composition of the aIS, we performed ultrastructural analysis of tangentially sectioned human retinas. We observed that each rod OS is accompanied by an adjacent aIS (Fig. 1c). At its base, the aIS is about as wide as the OS and contains several mitochondria, just as the rest of the IS (Example 1 in Fig. 1c). As the aIS extends along the OS, it narrows (Example 2) and eventually becomes devoid of mitochondria (Example 3). We also noticed that the cytoplasmic space surrounding the mitochondria contains densely packed filamentous material. When examined at higher magnification, these filaments were recognized to be longitudinally arranged microtubules (Fig. 2a). Their identity was confirmed by immunostaining of human rods with antibodies against β-tubulin (Fig. 2b), which revealed two microtubular structures emanating from the distal end of each rod IS. One of these structures is the ciliary axoneme, as evidenced by its origination from the area labeled for GPR98, an Usher protein located within the periciliary membrane surrounding the axoneme, whereas the other microtubular structure corresponds to the aIS. This is in contrast to the pattern of tubulin staining in mammalian species lacking the aIS, which yields staining of only the ciliary axoneme (e.g., refs. ^12,13^). These observations suggest that the aIS is fortified by a dense microtubule-based cytoskeleton, as illustrated in Fig. 2c.

**Fig. 3:**
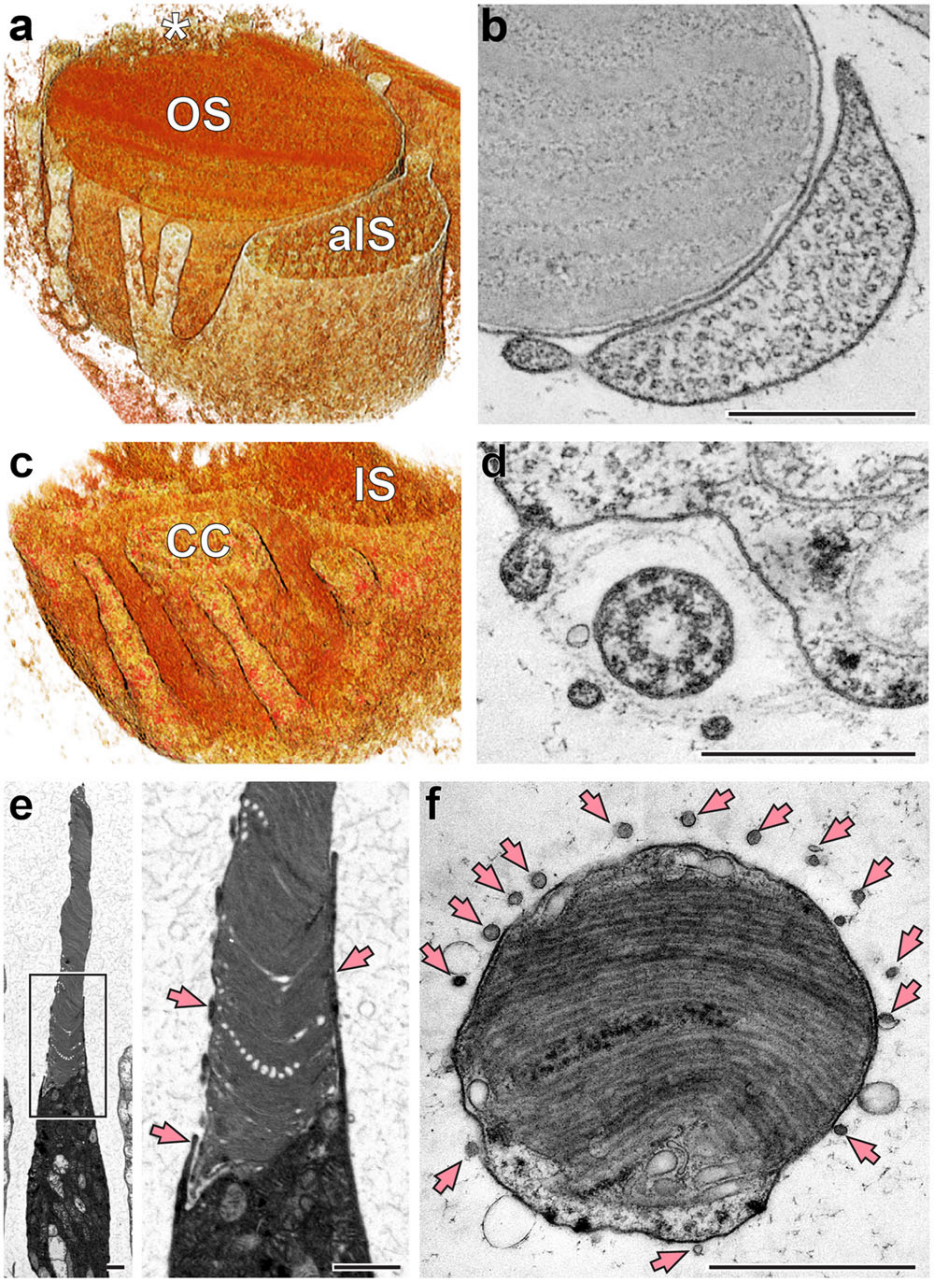
Rod outer segments are surrounded by microtubule-containing inner segment protrusions. **a** Rendering of a tomogram (Supplementary Movie 1) obtained from a rod within a tangential section of human retina. Asterisk indicates the ciliary axoneme. **b** A *z*-section of the same tomogram that captures a microtubule-enriched process at the level of its bifurcation from the aIS. Pixel size: 3.0 nm. **c**, **d** Rendering (**c**) and a *z*-section (**d**) of a tomogram (Supplementary Movie 3) obtained from a rod within a tangential section of human retina. Pixel size in (**d**): 2.1 nm. **e** TEM image of a cone from longitudinally sectioned human retina. The boxed area in the left panel is magnified to the right. **f** TEM image of a cone from tangentially sectioned human retina. Pink arrows in (**e**) and (**f**) indicate calyceal processes surrounding a cone OS. Scale bars: 0.5 µm (**b**, **d**); 1 µm (**e**, **f**).

To further explore the architecture of the aIS, we employed 3-dimensional electron tomography (3D-ET). 3D-ET has two advantages over conventional TEM. It allows the analysis of much thicker tissue sections than with TEM (such as ~750 nm sections in our experiments) and overcomes the limited *z*-resolution of TEM. While the *z*-resolution of TEM is determined by section thickness (typically 50–70 nm), the spatial resolution of 3D-ET is nearly isotropic, ultimately providing detailed tissue reconstruction within nanometer resolution in all three dimensions. Representative tomograms of three rods are shown in Supplementary Movies 1–3. Each of these tomograms demonstrates that, in addition to the very large aIS, rod outer segments are surrounded by multiple inner segment protrusions of smaller diameter, most of which contain microtubules. Some of them branch off from the aIS structure, as visualized by a rendering of the tomogram from Supplementary Movie 1 spanning ~600 nm of the axial photoreceptor length (Fig. 3a). A single *z*-section at the level of this bifurcation is shown in Fig. 3b. Another example of multiple protrusions surrounding the outer segment, which contain microtubules, is shown in Supplementary Movie 2. Other protrusions emanate directly from the distal end of the IS in the area behind the connecting cilium (Supplementary Movie 3 and Fig. 3c, d).

### The accessory inner segment is distinct from calyceal processes

In some ways, the aIS and its branches resemble calyceal processes, which are microvilli-like extensions emanating from the IS of rods and cones and surrounding the OS in many species (reviewed in ref. ^5^). However, calyceal processes are supported by an actin network, not microtubules (e.g., refs. ^12,14^), which indicates that aIS and smaller microtubule-containing IS protrusions are not simply large calyceal processes but rather previously unrecognized types of structures observed thus far only in human rods.

In contrast to rods, we did not encounter the presence of aIS in human cones (Fig. 3e, f). Instead, proximal regions of cone OS are surrounded only by multiple thin microvilli-like extensions previously described as actin-enriched calyceal processes (e.g., ref. ^12^). This leads us to conclude that the aIS is a unique feature of human rods distinct from calyceal processes. In the next part of this study, we turned our attention to investigating the possible role of this unusual structure.

### The accessory inner segment is connected to the outer segment via electron-dense structures

**Fig. 4:**
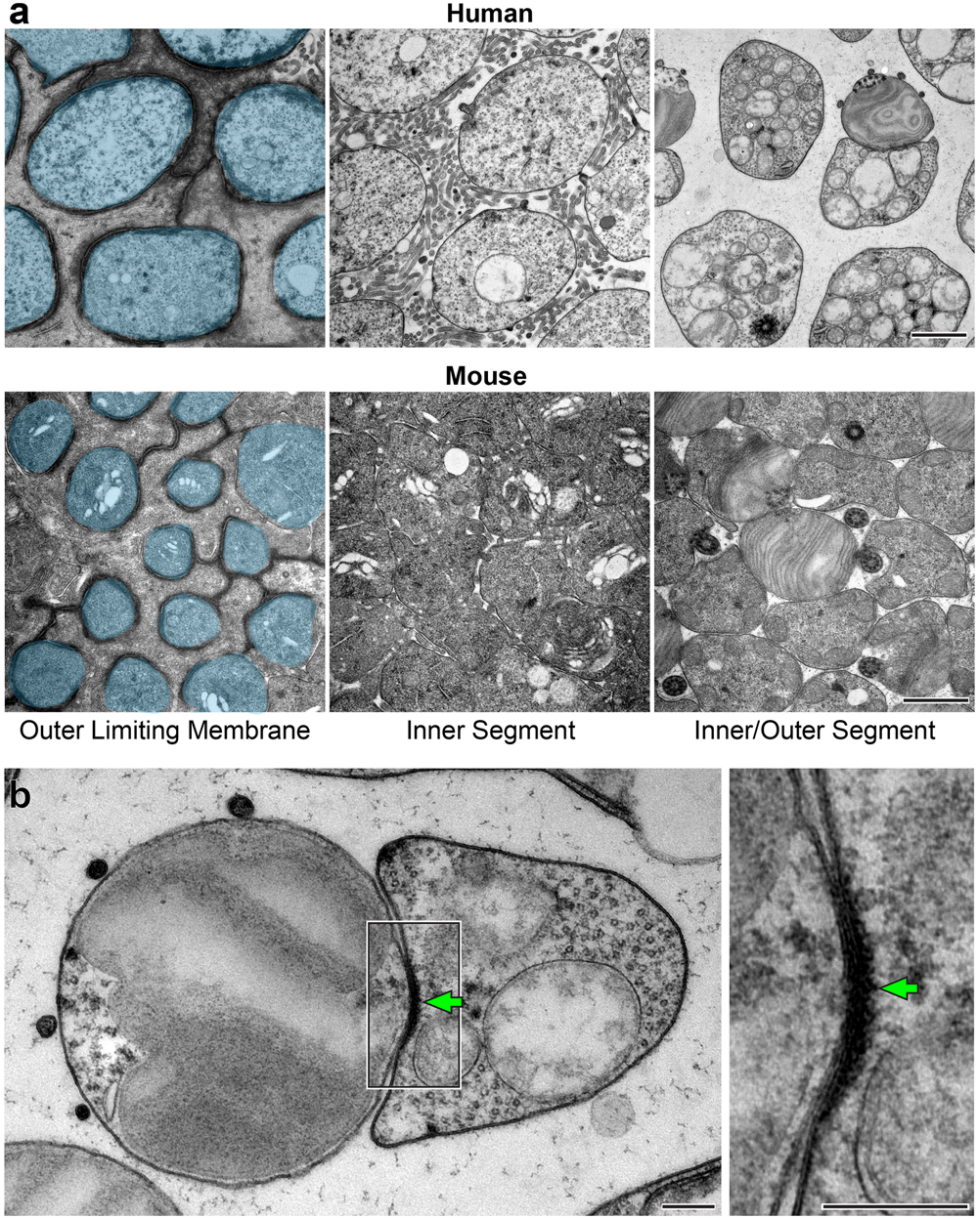
The accessory inner segment is connected to the outer segment via electron-dense structures. **a** TEM images of human and mouse retinas tangentially sectioned at different levels, as indicated. Photoreceptor cells in the left panels are pseudo-colored blue to distinguish them from Müller glial cells. **b** TEM image of a rod from tangentially sectioned human retina. The boxed area on the left is magnified on the right. Green arrows indicate an electron-dense structure located between aIS and OS. Scale bars: 1 µm (**a**); 0.2 µm (**b**).

The functional role of the aIS is not immediately clear. One consideration is that the spacing of human extrafoveal photoreceptors in the IS/OS region is sparse relative to many non-primate species, including mice (e.g., ref. ^15^). This sparse organization can be appreciated in Fig. 1c and is further visualized by imaging human retinas tangentially sectioned at different planes, ranging from the outer limiting membrane (OLM; located at the proximal base of the IS) to the IS/OS junction (Fig. 4a). Around the OLM, individual human photoreceptor cells are fully separated by Müller glial cells, with which they form adherens junctions. Immediately above the OLM, endfeet microvilli of Müller glial cells occupy most of the space between photoreceptors. At the OS base, photoreceptor cells remain separated to a similar degree by an abundant amount of interphotoreceptor matrix material. In contrast, mouse photoreceptors are densely packed at all levels (Fig. 4a).

Considering this spatial arrangement of human rods, we speculate that the functional role of the aIS is to provide physical support to the sparsely spaced OS. This hypothesis presumes that the OS and the adjacent aIS are not merely juxtaposed but also physically connected. Therefore, we sought to investigate whether there are any structural features at their interface. Using TEM and 3D-ET, we identified electron-dense structures that span the plasma membranes of OS and aIS and bring these membranes remarkably close to one another (Fig. 4b and Supplementary Movie 4). These electron-dense structures could serve to maintain the close apposition between aIS and OS, which would be necessary for the aIS to provide physical support to the OS.

## Discussion

The central finding of this study is that human rod photoreceptors possess a unique structure consisting of a large protrusion of the IS reinforced by an extensive microtubule-based network and connected to the OS through distinct intermembrane adhesions. We termed this structure the accessory *inner* segment (Fig. 2c), inspired by the term accessory *outer* segment, used to describe a sub-compartment of the teleost fish OS containing the ciliary axoneme^16,17^. In contrast, the aIS is an extension of the IS containing non-ciliary microtubules. To the best of our knowledge, the presence of such large microtubule-enriched structures has not been reported in either humans or other species, including the thoroughly characterized macaque monkeys^12^. Whether aIS are present in rods of other non-human primates, particularly apes, is an interesting evolutionary question warranting future studies. Furthermore, while we qualitatively observed the presence of the aIS in peripheral and central retina, a thorough quantitative analysis of the aIS across retinal regions of both young and aged donors is the subject of ongoing investigation.

While it is conceivable that aIS merely fill the space between the sparsely packed human photoreceptors, the presence of unique electron-dense structures juxtaposing the membranes of the OS and the aIS argues otherwise. Combined with the fact that the aIS contains an extensive microtubule network, the presence of these adhesions suggests that the most plausible function of the aIS is to provide mechanical support to the OS. The significance of such support remains to be determined, as well as whether a disruption of this structure or loss of its association with the OS may affect normal photoreceptor function. It is well-documented that many hereditary visual disorders affecting human photoreceptors are either not phenocopied in mice bearing the same mutations (e.g., ref. ^18^) or the respective genes are not even present in mice (e.g., ref. ^19^). It is tempting to speculate that at least some of these differences are explained by the lack of the aIS in photoreceptors of commonly used animal models.

## Methods

### Human donor eye tissue

Enucleated human globes were obtained by Miracles in Sight (Winston-Salem, NC) and distributed by the BioSight Tissue Repository and Service Center (Duke University) under an approved Institutional Review Board protocol. Retinas used for electron microscopy analysis originated from a 61-year-old male and a 73-year-old female, whereas retinas used for immunofluorescence microscopy originated from a 63-year-old female. Only donors without medical history of ocular pathology were selected. All tissues were de-identified prior to being delivered to the laboratory. Whole globes were enucleated and kept on ice in a humidified chamber for 6–12 h until further processing.

### Animal husbandry

Animal maintenance and experiments were approved by the Institutional Animal Care and Use Committees at Duke University. We have complied with all relevant ethical regulations for animal use. WT mice (*Mus musculus*) were C57BL/6J (Jackson Labs stock #000664). We evaluated the retinas of a total of three (*n* = 3) healthy adult WT mice that were sex-randomized. No treatments or experimental groups were involved in this study; the sample size was deemed sufficient to reflect the anatomy of WT retinas based on our previous experience; there were no inclusion/exclusion criteria; no retinas were excluded from analysis; no confounders were identified; blinding was not applicable; outcome measures included TEM analyses of retinal anatomy; the study did not include statistical analyses.

### Transmission electron microscopy (TEM)

For humans, enucleated globes were fixed 6–7 h after death in a solution of 2% paraformaldehyde, 2% glutaraldehyde and 0.05% calcium chloride in 50 mM MOPS (pH 7.4) for 1 week. Following the removal of the anterior segment and vitreous humor, 12 mm punches of retina were obtained from the retinal mid-periphery. For longitudinal sections, tissue was embedded in 2.5% low melting point agarose (Precisionary) and cut into 200 µm thick slices on a Vibratome (VT1200S; Leica). For tangential sections, dissected tissue was processed whole.

For mice, anesthetized animals were transcardially perfused with a solution of 2% paraformaldehyde, 2% glutaraldehyde and 0.05% calcium chloride in 50 mM MOPS (pH 7.4). Enucleated eyes were fixed for an additional 2 h in the same fixation solution at RT. Following the removal of the cornea and lens, agarose sections from eyecups were cut as described above.

Resulting agarose sections or tissues were stained with 1% tannic acid (Electron Microscopy Sciences) and 1% uranyl acetate (Electron Microscopy Sciences), gradually dehydrated with ethanol and infiltrated and embedded in Spurr’s resin (Electron Microscopy Sciences). Then, 70 nm sections were cut, placed on copper grids and counterstained with 2% uranyl acetate and 3.5% lead citrate (19314; Ted Pella). The samples were imaged on a JEM-1400 electron microscope (JEOL) at 60 kV with a digital camera (BioSprint; AMT). Image analysis and processing were performed with ImageJ.

### Three-dimensional intermediate high voltage electron microscopic tomography (3D-ET) of thick sections

Either 250 or 750 nm thick retinal sections were cut and placed on 50 nm Luxel film slot grids. Grids were glow-discharged on both sides, and a mixture of 10 nm, 20 nm and 60 nm gold particles were deposited on the sample surfaces to serve as fiducial markers. 3D-ET was conducted on a Titan Halo (FEI, Hillsboro, OR, USA) operating at 300 kV in TEM mode for 250 nm sections or in STEM mode for 750 nm sections. A four-tilt series data acquisition scheme previously described^20^ was followed in which the specimen was tilted from −60° to +60° every 0.25° at four evenly distributed azimuthal angle positions. Images were collected on an 8k×8k direct detector (DE64; Direct Electron, San Diego, CA, USA) in TEM mode or with a high-angle annular dark field (HAADF) detector in STEM mode. The final volumes were generated using an iterative reconstruction procedure^20^. 3dmod and ImageJ were used for image analysis, and Amira was used for volume rendering.

### Immunofluorescence microscopy

Enucleated human globes were dissected 12 h after death. Following the removal of the anterior segment and vitreous humor, the posterior segment was cut into several pieces, and retinal tissue carefully removed and fixed in 4% paraformaldehyde in PBS for 1 h. Tissue was embedded in low melting point agarose and sectioned using a Vibratome at 100 µm thickness. Sections were blocked in PBS containing 7% donkey serum and 0.5% Triton X-100 for 30 min at RT and incubated with primary antibodies against β-tubulin (1:500; T4026; Millipore-Sigma) and GPR98 (1:200; PA5-84761; Thermo Fisher Scientific) overnight at 4 °C. Sections were washed and incubated with secondary donkey anti-mouse and anti-rabbit antibodies conjugated to Alexa Fluor 488 or 568 (1:1000; A21202 and A10042, respectively; Thermo Fisher Scientific) for 2 h at RT. Sections were washed and mounted onto slides with Shandon Immu-Mount (9990402; Thermo Fisher Scientific). Images were acquired using a confocal microscope (Eclipse 90i and A1 confocal scanner; Nikon) with a 60× objective (1.49 NA Plan Apochromat VC; Nikon) and NIS-Elements software (Nikon). Optical *z*-sections were collected at a resolution of 1024 × 1024 pixels with a step size of 0.15 μm. 3D deconvolution was performed in NIS-Elements, and deconvolved *z*-stacks were processed using ImageJ. Images are shown as maximum intensity *z*-projections over a depth of 1.5 μm.

### Reporting summary

Further information on research design is available in the Nature Portfolio Reporting Summary linked to this article.

## Supplementary information

Description of Additional Supplementary Files

Supplementary Movie 1

Supplementary Movie 2

Supplementary Movie 3

Supplementary Movie 4

Reporting Summary

## Data Availability

The datasets generated during and/or analyzed during the current study are available from the corresponding author upon reasonable request.
