# Supplementary material for: Unique ultrastructural organization of human rod photoreceptors
Source: Commun Biol. 2025 Jan 16;8:63. doi: 10.1038/s42003-025-07473-6 (PMC11739419; doi:10.1038/s42003-025-07473-6)
Supplement: Supplementary file 1 — Description of Additional Supplementary Files [file 42003_2025_7473_MOESM1_ESM.pdf]

Description of Additional Supplementary Files

File name: Supplementary Movie 1.

Description: Reconstructed tomogram of a human rod outer segment and corresponding accessory inner segment – Example 1. Shown is a 579 nm fragment of a 750- nm-thick retinal section with an isotropic resolution of 3.0 nm.

File name: Supplementary Movie 2.

Description: Reconstructed tomogram of a human rod outer segment and corresponding accessory inner segment – Example 2. Shown is a 141 nm fragment of a 250- nm-thick retinal section with an isotropic resolution of 1.4 nm.

File name: Supplementary Movie 3.

Description: Reconstructed tomogram of presumed calyceal processes. Shown is a 481 nm fragment of a 750-nm-thick retinal section with an isotropic resolution of 2.1 nm.

File name: Supplementary Movie 4.

Description: Reconstructed tomogram of electron-dense structures between the outer segment and accessory inner segment. Shown is a 104 nm fragment of a 250-nm-thick retinal section with an isotropic resolution of 0.56 nm
