# Supplementary material for: Unique ultrastructural organization of human rod photoreceptors
Source: Commun Biol. 2025 Jan 16;8:63. doi: 10.1038/s42003-025-07473-6 (PMC11739419; doi:10.1038/s42003-025-07473-6)
Supplement: Supplementary file 6 — Reporting Summary [file 42003_2025_7473_MOESM6_ESM.pdf]

Corresponding author(s): Oleg Alekseev, Tylor R. Lewis, Vadim Y Arshavsky

Last updated by author(s): Dec 31, 2024

Reporting Summary

Nature Portfolio wishes to improve the reproducibility of the work that we publish. This form provides structure for consistency and transparency in reporting. For further information on Nature Portfolio policies, see our Editorial Policies and the Editorial Policy Checklist.

Statistics

For all statistical analyses, confirm that the following items are present in the figure legend, table legend, main text, or Methods section.

n/a	Confirmed	
☐	☒	The exact sample size (*n*) for each experimental group/condition, given as a discrete number and unit of measurement
☒	☐	A statement on whether measurements were taken from distinct samples or whether the same sample was measured repeatedly
☒	☐	The statistical test(s) used AND whether they are one- or two-sided<br>*Only common tests should be described solely by name; describe more complex techniques in the Methods section.*
☒	☐	A description of all covariates tested
☒	☐	A description of any assumptions or corrections, such as tests of normality and adjustment for multiple comparisons
☒	☐	A full description of the statistical parameters including central tendency (e.g. means) or other basic estimates (e.g. regression coefficient) AND variation (e.g. standard deviation) or associated estimates of uncertainty (e.g. confidence intervals)
☒	☐	For null hypothesis testing, the test statistic (e.g. *F*, *t*, *r*) with confidence intervals, effect sizes, degrees of freedom and *P* value noted<br>*Give P values as exact values whenever suitable.*
☒	☐	For Bayesian analysis, information on the choice of priors and Markov chain Monte Carlo settings
☒	☐	For hierarchical and complex designs, identification of the appropriate level for tests and full reporting of outcomes
☒	☐	Estimates of effect sizes (e.g. Cohen's *d*, Pearson's *r*), indicating how they were calculated

Our web collection on statistics for biologists contains articles on many of the points above.

Software and code

Policy information about availability of computer code

Data collection	NIS-Elements
Data analysis	NIH ImageJ, 3dmod, Amira, NIS-Elements

For manuscripts utilizing custom algorithms or software that are central to the research but not yet described in published literature, software must be made available to editors and reviewers. We strongly encourage code deposition in a community repository (e.g. GitHub). See the Nature Portfolio guidelines for submitting code & software for further information.

Data

Policy information about availability of data

All manuscripts must include a data availability statement. This statement should provide the following information, where applicable:

- Accession codes, unique identifiers, or web links for publicly available datasets
- A description of any restrictions on data availability
- For clinical datasets or third party data, please ensure that the statement adheres to our policy

All data are contained in the manuscript and supplementary information.

Research involving human participants, their data, or biological material

Policy information about studies with human participants or human data. See also policy information about sex, gender (identity/presentation), and sexual orientation and race, ethnicity and racism.

Reporting on sex and gender	Human retinas were obtained from male and female donors.
Reporting on race, ethnicity, or other socially relevant groupings	These data were not available.
Population characteristics	Based on the limited medical record data that accompanied donor tissues, the three donors were a 61-year-old male, a 73-year-old female, and a 63-year-old female. Donors did not have prior history of any retinal conditions.
Recruitment	Eye tissues were obtained postmortem from organ donors.
Ethics oversight	Duke University IRB.

Note that full information on the approval of the study protocol must also be provided in the manuscript.

Field-specific reporting

Please select the one below that is the best fit for your research. If you are not sure, read the appropriate sections before making your selection.

☒ Life sciences ☐ Behavioural & social sciences ☐ Ecological, evolutionary & environmental sciences

For a reference copy of the document with all sections, see nature.com/documents/nr-reporting-summary-flat.pdf

Life sciences study design

All studies must disclose on these points even when the disclosure is negative.

Sample size	This study examined retinas obtained from three different donors.
Data exclusions	No data were excluded from the analyses.
Replication	All attempts at replication were successful.
Randomization	This was an phenomenological study that did not require randomization.
Blinding	This was an phenomenological study that did not require blinding.

Reporting for specific materials, systems and methods

We require information from authors about some types of materials, experimental systems and methods used in many studies. Here, indicate whether each material, system or method listed is relevant to your study. If you are not sure if a list item applies to your research, read the appropriate section before selecting a response.

Materials & experimental systems

n/a	Involved in the study	
☐	☒	Antibodies
☒	☐	Eukaryotic cell lines
☒	☐	Palaeontology and archaeology
☐	☒	Animals and other organisms
☒	☐	Clinical data
☒	☐	Dual use research of concern
☒	☐	Plants

Methods

n/a	Involved in the study	
☒	☐	ChIP-seq
☒	☐	Flow cytometry
☒	☐	MRI-based neuroimaging

Antibodies

Antibodies used	Primary antibodies: β-tubulin (1:500; T4026; Millipore-Sigma), GPR98 (1:200; PA5-84761; Thermo Fisher Scientific). Secondary antibodies: donkey anti-mouse and anti-rabbit antibodies conjugated to Alexa Fluor 488 or 568 (1:1000; A21202 and A10042, respectively; Thermo Fisher Scientific).

Validation	All antibodies used in this study were extensively validated in the literature, by the manufacturers, and in our lab in prior experiments.

Animals and other research organisms

Policy information about studies involving animals; ARRIVE guidelines recommended for reporting animal research, and Sex and Gender in Research

Laboratory animals	Wild type mice (Mus musculus) were C57BL/6J (Jackson Labs stock #000664).
Wild animals	This study did not involve wild animals.
Reporting on sex	All experiments were performed with animals of randomized sex.
Field-collected samples	This study did not involve samples collected from the field.
Ethics oversight	Duke University IACUC.

Note that full information on the approval of the study protocol must also be provided in the manuscript.

Plants

Seed stocks	N/A
Novel plant genotypes	N/A
Authentication	N/A
